# Editorial: Artificial intelligence based computer-aided diagnosis applications for brain disorders from medical imaging data

**DOI:** 10.3389/fnins.2023.998818

**Published:** 2023-01-31

**Authors:** Ahmed Shalaby, Ahmed Soliman, Safa Elaskary, Ahmed Refaey, Mohamed Abdelazim, Fahmi Khalifa

**Affiliations:** ^1^Lyda Hill Department of Bioinformatics, UT Southwestern Medical Center, Dallas, TX, United States; ^2^Computer Engineering Department, Mansoura University, Mansoura, Egypt; ^3^Department of Biomedical Equipment and Technology, Applied Health Sciences, Pharos University, Alexandria, Egypt; ^4^School of Engineering and Physical Sciences University of Guelph, Guelph, ON, Canada; ^5^Electronics and Communications Engineering, Mansoura University, Mansoura, Egypt; ^6^Electrical and Computer Engineering, Morgan State University, Baltimore, MD, United States

**Keywords:** brain disorder, artificial intelligence, autism, Alzheimer's disease, Schizophrenia, MRI, DTI

There has been an exponential growth recently in research projects studying the etiology and mechanisms of several brain disorders, such as autism, Multiple Sclerosis (MS), Dementia, Alzheimer's Disease (AD), Gliomas, Schizophrenia, and Epilepsy. In recent years, the utility of artificial intelligence (AI) has been explored in a variety of research arenas including the development of modern computer-aided diagnosis (CAD) systems. The use of medical imaging and the characteristic examples provided by medical experts in AI-based CADs is a growing field with a goal of more accurate extraction of reliable diagnostic cues to eventually help physicians provide more appropriate and personalized treatments. For instance, texture analyses of the white matter on brain T2-weighted magnetic resonance imaging (MRI) can help in diagnosing MS. In addition, AI-based CADs will facilitate the interpretation and utilization of all available data, mitigating the overwhelming manual assessment, and make it practical in daily clinical practice.

Traditional machine learning (ML)-based CAD systems employ many learning techniques that are often tailored to a specific application and usually need a lot of tuning and even fail if tested outside the training data sets. Advances in AI techniques, particularly, end-to-end deep learning, combined with recent progress in neuroimaging technologies (e.g., diffusion-weighted MRI and other modalities for imaging the brain and the nervous system) have created exciting new opportunities for both enhancing traditional ML methods and applying new prospective ones to predict or provide better diagnosis of brain diseases.

The focus of this Research Topic is on the recent AI-based CAD systems for the analysis of medical imaging data from patients with brain disorders such as: Schizophrenia, Dementia, Alzheimer's, etc. The target audience for this Research Topic includes engineering and medical school professors; graduate and undergraduate college students in engineering and applied science departments; medical students; engineers working for medical companies; researchers in industry, academia, and health scientists; medical doctors such as radiologists; as well as healthcare professionals including radiology technologists and medical physicists.

The topic consists of 13 accepted research articles that deal with artificial intelligence/ML algorithms to investigate different brain problems. A brief summary of each article is provided below.

In Agostinho et al., a data integration approach for the comparison between the integration of structure MR and Diffusion Tensor Imaging (DT) vs. MR combined with Amyloid Positron Emission Tomography (PET) in detecting Alzheimer's disease (AD). Their idea is based on the unique combination of the three common imaging modalities, currently used in AD classification, and aim to explore the effects of all possible combinations between them. Furthermore, they want to evaluate if the combination of structural magnetic resonance imaging (sMRI) with diffusion tensor imaging (DTI) can achieve a comparable performance as sMRI combined with positron emission tomography (PET). These evaluations were performed by creating support vector machine (SVM) models for each modality (sMRI, DTI, PET), which were subsequently combined using a special ensemble technique. Wu, Dong, Zhang et al. propose a framework for federated morphometry feature selection (FMFS) model for Hippocampal morphometry associated Beta-Amyloid and Tau pathology. Amyloid-β (Aβ) plaques and tau protein tangles in the brain are now widely recognized as the defining hallmarks of AD, followed by structural atrophy detectable on brain magnetic resonance imaging (MRI) scans. One of the particular neurodegenerative regions is the hippocampus to which the influence of Aβ/tau on has been one of the researches focusing on the AD pathophysiological progress. The FMFS uses measured positron PET data to examine the subtle aspects of hippocampal morphometry that are associated with Aβ/tau burden in the brain. All the results indicate that FMFS is an efficient and effective tool to reveal associations between Aβ/tau burden and hippocampal morphometry.

In another research problem, diabetes with high blood glucose levels may damage the brain nerves and thus increase the risk of dementia. Previous studies have shown that dementia can be reflected in altered brain structure, facilitating computer-aided diagnosis of brain diseases based on sMRI. However, type 2 diabetes mellitus (T2DM)-mediated changes in the brain structures have not yet been studied, and only a few studies have focused on the use of brain MRI for automated diagnosis of T2DM. Hence, identifying MRI biomarkers is essential to evaluate the association between changes in brain structure and T2DM as well as cognitive impairment (CI). The study by Chen Y. et al. aims to investigate four methods to extract features from MRI, characterize imaging biomarkers, as well as identify subjects with T2DM and CI.

In a study by Wu, Dong, Gui et al., brain Amyloid is predicted using multivariate morphometry statistics, sparse coding, and correntropy. The authors optimize the objective function of the Patch Analysis-based Surface Correntropy-induced Sparse-coding and Max-Pooling (PASCS-MP) system by introducing correntropy measure and propose an improved sparse coding, dubbed as the PASCS-MP method. PASCS-MP does not only take the advantage of the computational efficiency of PASS-MP in its new optimization strategy, but also effectively reduces the negative influence of non-Gaussian noise in the data, which tremendously improves the prediction accuracy. PASCS-MP is an unsupervised learning method to generate a low-dimensional representation for each sample. They leverage the novel PASCS-MP method on MMS to further explore hippocampal morphometry differences for the following contrasts at the individual subject level: (1) Aβ positive individuals with mild cognitive impairment (Aβ+ MCI) vs. Aβ negative individuals with mild cognitive impairment (Aβ- MCI) from ADNI, and (2) Aβ positive CU subjects (Aβ+ CU from ADNI and OASIS) vs. Aβ negative CU subjects (Aβ- CU from ADNI and OASIS). Authors apply the proposed PASCS-MP and a binary random forest classifier to classify individuals with different Aβ status. They test their method in two independent cohorts, 841 subjects from the Alzheimer's Disease Neuroimaging Initiative (ADNI) and 260 subjects from the Open Access Series of Imaging Studies (OASIS). Experimental results suggest that the proposed PASCS-MP method and MMS can discriminate Aβ positivity in people with mild cognitive impairment (MCI) [Accuracy (ACC) = 0.89 (ADNI)] and in cognitively unimpaired (CU) individuals [ACC = 0.79 (ADNI) and ACC = 0.81 (OASIS)]. These results compare favorably relative to measures derived from traditional algorithms, including hippocampal volume and surface area, shape measures based on spherical harmonics (SPHARM) and prior PASS-MP methods.

ML methods have been explored by Zang et al. to study the effects of brain atlases on the discrimination of Schizophrenia (SZ). They collected MRI data for 61 first-episode SZ patients (FESZ), 79 chronic SZ patients (CSZ) and 205 normal controls (NC) and calculated four MRI measurements, including regional gray matter volume (GMV), regional homogeneity (ReHo), amplitude of low-frequency fluctuation and degree centrality. Authors systematically analyzed the performance of two classifications (SZ vs. NC; FESZ vs. CSZ) based on the combinations of three brain atlases, five classifiers, two cross validation methods and three dimensionality reduction algorithms. The results showed that the groupwise whole-brain atlas with 268 ROIs outperformed the other two brain atlases. In addition, the leave-one-out cross validation was the best cross validation method to select the best hyperparameter set, but the classification performances by different classifiers and dimensionality reduction algorithms were quite similar. In another paper for classifying SZ by combination of brain effective and functional connectivity, Zhao et al. collected event-related potentials from 45 SZ patients and 30 healthy controls (HCs) during a learning task. Then, they used a combination of partial directed coherence (PDC) effective and phase lag index (PLI) functional connectivity as features to train a support vector machine classifier with leave-one-out cross-validation for classification of SZ from HCs. Experimental results indicated that the classification performance (accuracy = 95.16%, specificity = 94.44%, and sensitivity = 96.15%) was obtained when the combination of functional and effective connectivity features was used, and the corresponding optimal feature number was 15, which included 12 PDC and three PLI connectivity features. The selected effective connectivity features were mainly located between the frontal/temporal/central and visual/parietal lobes, and the selected functional connectivity features were mainly located between the frontal/temporal and visual cortexes of the right hemisphere. Although the obtained results are promising, the use of low number of patients limits the ability to draw a generic conclusion and there is a need for more comprehensive study on larger dataset. Additionally, recent research showed detecting SZ brain MRIs is a great challenge (Oh et al., 2020), thus additional tests and brain images should be combined for comprehensive evaluation.

Leng et al. presented a study that (1) explores the changes in topological properties of static and dynamic brain functional networks after nasopharyngeal carcinoma (NPC) radiotherapy (RT) using rs-fMRI and graph theoretical analysis, (2) explores the correlation between cognitive function and changes in brain function, and (3) add to the understanding of the pathogenesis of radiation brain injury (RBI). The disruption of static and dynamic functional network stability, reduced network efficiency and reduced functional connectivity may be potential biomarkers of RBI. The findings may provide new insights into the pathogenesis of RBI from the perspective of functional networks.

Fletcher et al. laid out the methods by which they have achieved consistently high quality, high throughput computation of intra-cranial segmentation from whole brain MRIs. This task is essential, but typically time-consuming bottleneck for brain image analysis. They refer to this output as “production-level” because it is suitable for routine use in processing pipelines. Based on training and testing of the developed method with an extremely large archive of structural images, their segmentation algorithm performs uniformly well over a wide variety of separate national imaging cohorts, giving Dice metric scores exceeding those of other recent deep learning brain extractions. Authors describe the components involved to achieve this performance, including size, variety and quality of ground truth, and appropriate neural net architecture. They demonstrate the crucial role of appropriately large and varied datasets, suggesting a less prominent role for algorithm development beyond a threshold of capability.

A gradient-based features extracted from sMRI images are used to depict the subtle changes within brains of patients with gliomas. Based on the gradient features, Chen T. et al. proposed a novel two-phase classification framework for detection and grading of gliomas. In the first phase, the probability of each local feature being related to different types (e.g., diseased or healthy for detection, and benign or malignant for grading) was calculated. Then the high-level feature representing the whole MRI image was generated by concatenating the membership probability of each local feature. In the second phase, the supervised classification algorithm was used to train a classifier based on the high-level features and patient labels of the training subjects. Authors applied this framework on the brain imaging data collected from Zhongnan Hospital of Wuhan University for glioma detection, and the public TCIA datasets including glioblastomas (WHO IV) and low-grade gliomas (WHO II and III) data for glioma grading. The experimental results showed that the gradient-based classification framework could be a promising tool for automatic diagnosis of brain tumors.

Liu et al. used ML and DTI to predict individual severity of blepharospasm. Accumulating DTI evidence suggests that white matter abnormalities evaluated by local diffusion homogeneity (LDH) or fractional anisotropy (FA) occur in patients with blepharospasm (BSP), both of which are significantly correlated with disease severity. However, whether the individual severity of BSP can be identified using these DTI metrics remains unknown. In this article, authors aimed to investigate whether a combination of machine learning techniques and LDH or FA can accurately identify the individual severity of BSP. Forty-one patients with BSP were assessed using the Jankovic Rating Scale and DTI. The patients were assigned to non-functionally and functionally limited groups according to their Jankovic Rating Scale scores. An ML scheme consisting of beam search and SVMs were designed to identify non-functionally vs. functionally limited outcomes, with the input features being LDH or FA in 68 white matter regions. The proposed machine learning scheme with LDH or FA yielded an overall accuracy of 88.67 vs. 85.19% in identifying non-functionally limited vs. functionally limited outcomes. The scheme also identified a sensitivity of 91.40 vs. 85.87% in correctly identifying functionally limited outcomes, a specificity of 83.33 vs. 83.67% in accurately identifying non-functionally limited outcomes, and an area under the curve of 93.7 vs. 91.3%. These findings suggest that a combination of LDH or FA measurements and a sophisticated machine learning scheme can identify the individual disease severity in patients with BSP. More testing and investigation of the presented approach using larger dataset to show the clinical promise.

Multimodal heterogeneous data, such as sMRI, PET, and cerebrospinal fluid (CSF), are effective in improving the performance of automated dementia diagnosis by providing complementary information on degenerated brain disorders, such as Alzheimer's prodromal stage, i.e., mild cognitive impairment. Effectively integrating multimodal data has remained a challenging problem, especially when these heterogeneous data are incomplete due to poor data quality and patient dropout. Besides, multimodal data usually contain noise information caused by different scanners or imaging protocols. The existing methods usually fail to well-handle these heterogeneous and noisy multimodal data for automated brain dementia diagnosis. To this end, Dong et al. proposed a high-order Laplacian regularized low-rank representation method for dementia diagnosis using block-wise missing multimodal data. The proposed method was evaluated on 805 subjects (with incomplete MRI, PET, and CSF data) from Alzheimer's Disease Neuroimaging Initiative (ADNI). Experimental results suggest the effectiveness of their presented method in three tasks of brain disease classification, compared with the state-of-the-art methods.

Hu et al. presented a paper that classifies frontotemporal dementia (FTD) and AD using DL-based classification in addition to voxel-based visualization. FTD and AD have overlapping symptoms, and accurate differential diagnosis is important for targeted intervention and treatment. Previous studies suggest that the deep learning (DL) techniques have the potential to solve the differential diagnosis problem of FTD, AD and normal controls (NCs), but its performance is still unclear. In addition, existing DL-assisted diagnostic studies still rely on hypothesis-based expert-level preprocessing. On the one hand, it imposes high requirements on clinicians and data themselves; On the other hand, it hinders the backtracking of classification results to the original image data, resulting in the classification results cannot be interpreted intuitively. In the presented study, a large cohort of 3D T1-weighted structural magnetic resonance imaging (MRI) volumes (*n* = 4,099) was collected from two publicly available databases, i.e., the ADNI and the NIFD. Authors trained a DL-based network directly based on raw T1 images to classify FTD, AD and corresponding NCs. And they evaluated the convergence speed, differential diagnosis ability, robustness, and generalizability under nine different scenarios. The proposed network yielded an accuracy of 91.83% based on the most common T1-weighted sequence [magnetization-prepared rapid acquisition with gradient echo (MPRAGE)]. Experimental results demonstrated that DL-based networks have the ability to solve the enigma of differential diagnosis of diseases without any hypothesis-based preprocessing. Moreover, they may mine the potential patterns that may be different from human clinicians, which may provide new insight into the understanding of FTD and AD.

Finally, Sone and Beheshti presented a paper that reviews the clinical applications of ML models for brain imaging in epilepsy obtained from a PubMed database search in February 2021. The authors present an overview of typical neuroimaging modalities and ML models used in the epilepsy studies. Subsequently, they focused on the existing applications of ML models for brain imaging in epilepsy based on the following clinical aspects: (i) distinguishing individuals with epilepsy from healthy controls, (ii) lateralization of the temporal lobe epilepsy focus, (iii) the identification of epileptogenic foci, (iv) the prediction of clinical outcomes, and (v) brain-age prediction. They address the practical problems and challenges described in the literature and suggest some future research directions. Recent trends include the use of deep learning, multimodal imaging, and regression models, and additional investigations using unsupervised clustering are desired. For better clinical applications, consistent methodologies must be developed and validated using standard and common benchmarks.

## Author contributions

All authors listed have made a substantial, direct, and intellectual contribution to the work and approved it for publication.
